# Prognostic value of PD-L1 expression on tumor cells combined with CD8+ TIL density in patients with locally advanced non-small cell lung cancer treated with concurrent chemoradiotherapy

**DOI:** 10.1186/s13014-019-1453-3

**Published:** 2020-01-02

**Authors:** Kathrin Gennen, Lukas Käsmann, Julian Taugner, Chukwuka Eze, Monika Karin, Olarn Roengvoraphoj, Jens Neumann, Amanda Tufman, Michael Orth, Simone Reu, Claus Belka, Farkhad Manapov

**Affiliations:** 1 Department of Radiation Oncology, University Hospital, LMU Munich, Marchioninistrasse 15, 81377 Munich, Germany; 2 Comprehensive Pneumology Center Munich (CPC-M), Member of the German Center for Lung Research (DZL), Munich, Germany; 3 German Cancer Consortium (DKTK), partner site Munich, Munich, Germany; 4 Institute of Pathology, Faculty of Medicine, LMU, Munich, Germany; 5 Department of Internal Medicine V, Thoracic Oncology Centre Munich, Division of Respiratory Medicine and Thoracic Oncology, LMU, Munich, Germany; 6 Department of Pathology, University Hospital Wuerzburg, Wuerzburg, Germany

**Keywords:** TILs, PDL1, Chemoradiotherapy, Prognostic factors, Checkpoint inhibition

## Abstract

**Background/aim:**

mmune checkpoint inhibition (CPI) has an increasing impact in the multimodal treatment of locally advanced non-small cell lung cancer (LA-NSCLC). Increasing evidence suggests treatment outcome depending on tumor cell PD-L1 expression. The purpose of this retrospective study was to investigate the prognostic value of PD-L1 expression on tumor cells in combination with CD8+ tumor stroma-infiltrating lymphocyte (TIL) density in inoperable LA-NSCLC treated with concurrent chemoradiotherapy (CRT).

**Patients and method:**

We retrospectively assessed clinical characteristics and initial tumor biopsy samples of 31 inoperable LA-NSCLC patients treated with concurrent CRT. Prognostic impact of tumor cell PD-L1 expression (0% versus ≥1%) and CD8+ TIL density (0–40% vs. 41–100%) for local control, progression-free (PFS) and overall survival (OS) as well as correlations with clinicopathological features were evaluated.

**Results:**

Median OS was 14 months (range: 3–167 months). The OS rates at 1- and 2 years were 68 and 20%. Local control of the entire cohort at 1 and 2 years were 74 and 61%. Median PFS, 1-year and 2-year PFS were 13 ± 1.4 months, 58 and 19%. PD-L1 expression < 1% on tumor cells was associated with improved OS, PFS and local control in patients treated with concurrent CRT. Univariate analysis showed a trend towards improved OS and local control in patients with low CD8+ TIL density. Evaluation of Tumor Immunity in the MicroEnvironment (TIME) appears to be an independent prognostic factor for local control, PFS and OS. The longest and shortest OS were achieved in patients with type I (PD-L1^neg^/CD8^low^) and type IV (PD-L1^pos^/CD8^low^) tumors (median OS: 57 ± 37 vs. 10 ± 5 months, *p* = 0.05), respectively.

**Conclusion:**

Assessment of PD-L1 expression on tumor cells in combination with CD8+ TIL density can be a predictive biomarker in patients with inoperable LA-NSCLC treated with concurrent CRT.

## Introduction

Lung cancer remains the leading cause of cancer-related mortality worldwide [1–3]. Locally-advanced non-small cell lung cancer (LA-NSCLC) represents as a heterogeneous disease including large tumor volume, extensive lymph node involvement, tumor-related atelectasis and infiltration of the thoracic wall, mediastinum and spine [4–6]. The majority of LA-NSCLC patients are inoperable and multimodal approaches are considered a cornerstone of treatment [7–10]. Historically, administering platinum-based chemotherapy concurrently to thoracic irradiation resulted in modest improvements of local control, metastasis-free and overall survival (OS) compared to radiotherapy alone [11, 12]. In the last years, the role of immune checkpoint inhibition (CPI) in the multimodal treatment of LA-NSCLC has evolved [9, 12]. In 2015, the first programmed cell death protein 1 (PD-1) inhibitor (nivolumab) was approved by the Food and Drug Administration (FDA) for advanced or metastatic NSCLC in the second-line setting following progression during or after platinum-based chemotherapy [13, 14]. Subsequently in 2016, the FDA approved monotherapy with the PD-1 inhibitor pembrolizumab in the first-line setting for patients with metastatic NSCLC with programmed cell death 1 ligand 1 (PD-L1) Tumor Proportion Score (TPS) ≥ 50% and expanded the indication in April 2019 based on the results of the KEYNOTE-042 trial for the first-line treatment of patients with stage III patients who are not candidates for surgical resection or definitive CRT or metastatic NSCLC with TPS ≥ 1% determined by an FDA-approved test. Patients’ tumors had no Epidermal Growth Factor Receptor (EGFR) or Anaplastic lymphoma kinase (ALK) genomic aberrations [15, 16].

The addition of pembrolizumab to chemotherapy resulted in significantly higher rates of response and longer PFS than chemotherapy alone in a phase 2 cohort of the KEYNOTE-021 trial [17] and the FDA granted accelerated approval in May, 2017. CPI and chemotherapy combination therapy was also tested in the first-line setting in the KEYNOTE-189 and KEYNOTE-407 trials for metastatic nonsquamous NSCLC without sensitizing EGFR or ALK mutations and squamous NSCLC, respectively [18, 19]. Both studies reporting significantly improved OS and progression-free survival (PFS) than chemotherapy alone. Furthermore, the IMpower150 trial demonstrated superior PFS and OS for carboplatin/paclitaxel, bevacizumab and the PD-L1 blocking antibody atezolizumab vs. carboplatin/paclitaxel and bevacizumab in metastatic nonsquamous NSCLC, regardless of PD-L1 status and EGFR or ALK genetic alteration status [20]. Both combinations have been approved by the FDA.

Vis-à-vis stage III NSCLC, as a result of the PACIFIC trial, maintenance treatment with PD-L1 inhibitor durvalumab after successful completion of platinum-based concurrent chemoradiotherapy (CRT) has demonstrated significantly improved PFS and OS and became a new standard of care in inoperable stage III NSCLC [8, 9]. Currently, predictors for response to CPI are unclear and potential biomarkers are under investigation including PD-L1 expression of tumor cells, tumor-infiltrating lymphocytes (TIL), T-effector-interferon-γ-associated gene expression and tumor mutational burden (TMB) [21–23]. High mutation load has been shown to correlate with an immunogenic tumor microenvironment with increased expression of tumor-specific neo-antigens that can be targeted by activated immune cells e.g. cytotoxic CD8+ TILs [24, 25].

Considering the importance of PD-L1 expression on tumor cells and CD8 TIL density in defining the tumor immune microenvironment, we aimed to study PD-L1 expression alone and in combination with CD8 TIL density with relation to clinicopathologic characteristics and survival in patients treated with concurrent CRT.

## Methods

### Patients and samples

This study included 31 patients who received concurrent CRT for locally advanced or metastatic NSCLC. From their medical records, we retrieved patients’ clinical data, such as sex, age, histologic type and grading, pack years and TNM stage (using the 8th UICC TNM Staging System of lung cancer). Evaluation of EGFR/ALK genomic aberrations was performed in nonsquamous metastatic patients and was negative. All patients were closely followed-up according to an in-house protocol - every 3 months in the first 2 years, every 6 months up to 5 years and afterwards once per year. Expert pathologists (J.N. and S.R.) re-reviewed hematoxylin-eosin–stained slides from all cases, and corresponding formalin-fixed, paraffin-embedded specimens and performed the immunohistochemical staining.

### Immunohistochemistry

**Fig. 1:**
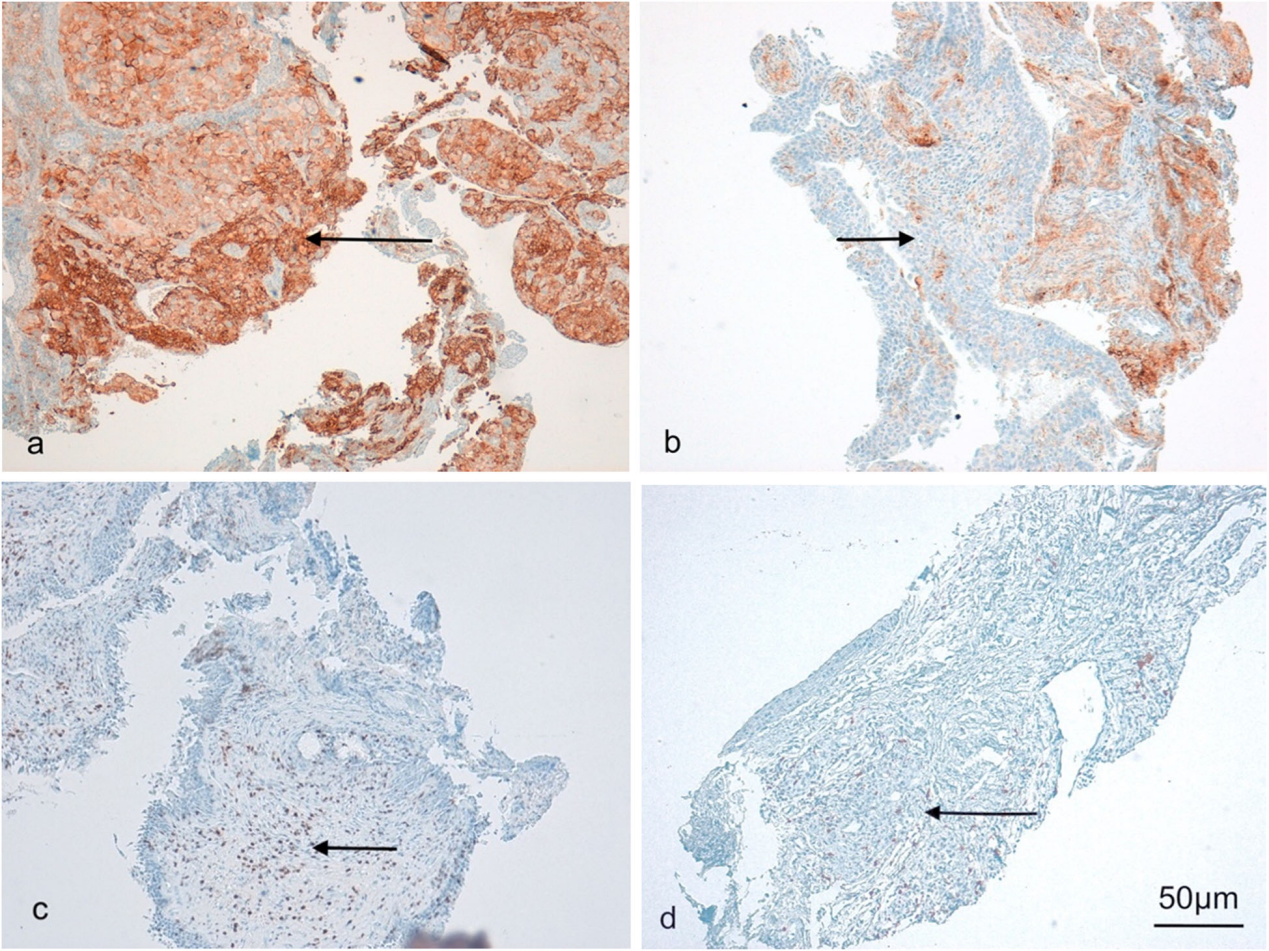
PD-L1 expression on tumor cells and CD8 expression on tumor infiltrating lymphocytes**.** Representative images under a microscope with a 10x enlargement of adenocarcinomas with positive (a, 80%) and negative (b, 0%) PD-L1 staining on tumor cells and adenocarcinomas with positive (c, 70%) and negative (d, 2%) CD8 staining on tumor infiltrating lymphocytes. The arrows indicate positive (**a**,**c**) or negative (**b**,**d**) staining

All immunohistochemical stainings were done on 5 μm whole standard tissue sections of formaldehyde-fixed paraffin-embedded tissue (FFPE) tumor samples (see Fig. 1). For the detection of PD-L1 prediluted PD-L1 rabbit monoclonal antibody (SP263; Ventana Medical Systems, Oro Valley, Arizona) was used as the primary antibody. Immunohistochemical staining for CD8 was carried out with an anti- CD8α mouse monoclonal antibody (C8/144B, Cell Marque, Rocklin, California, dilution 1:50) as the primary antibody. Both stainings were performed on a Ventana Benchmark Ultra autostainer using the UltraView diaminobenzidine kit (Ventana Medical Systems, Oro Valley, AZ).

### Assessment of PD-L1 expression

PD-L1 expression on tumor cells was measured quantitatively using an established immunohistochemistry assay (Ventana SP 263) which had been used recently published randomized phase III studies [8, 9, 26]. All of the stained sections were scored in five randomly selected areas containing tumor cells, which showed membranous and cytoplasmic staining. The percentage of positive tumor cells was graded on a scale of 0–2: 0 (< 1%), 1 (1–5%); 2 (> 5%). The intensity of staining was scored as follows: 0 (no staining), 1 (weak staining), 2 (moderate or strong staining). The H-score, ranging from 0 to 12, was calculated by multiplying the percentage of positive tumor cells by the intensity of staining on the tissue sections. The H-scores were categorized as follows: 0: negative (−), 1–4: weak positive (+), 5–8: moderately positive (+ +), 9–12: strong positive (+ + +).

### Assessment of CD8+ TIL density

Assessment of CD8+ TIL density was performed according to established breast cancer protocols [23]. In literature, common cut-off points ranged between 2.5 and 40% in order to differentiate between high and low CD8+ TIL density. In our study we divided the patient cohort in two subgroups (low and high density of CD8+ TILs: 0–40% vs. 41–100%).

### Assessment of tumor immunity in the MicroEnvironment (TIME)

Based on previous studies, four different types of tumour immune microenvironment have been identified according to PD-L1 expression of tumor cells and presence or absence of TILs in the tumor microenvironment [27, 28]. These included type I (PD-L1 − with no TILs indicating immune ignorance), type II (PD-L1 + with TILs implying adaptive immune resistance**)**, type III (PD-L1 − with TILs suggesting the role of other suppressor(s) in promoting immune tolerance) and type IV (PD-L1 + with no TILs indicating intrinsic induction). All patients were stratified according to TIME classification and TIME subgroups were evaluated for prognostic outcome, local control, PFS and OS.

### Statistical analysis

Each clinicopathologic characteristic was evaluated using Pearson’s chi-squared test or Fisher’s exact test (categorical variables). OS was measured from the date of the initial diagnosis until the date of death. The Kaplan-Meier method and log-rank test were applied to assess OS. In multivariate analysis, the Cox regression proportional hazard model was used to assess the clinicopathologic characteristics significantly related to OS with HRs and 95% CIs. A two-sided *p* value of ≤0.05 was considered statistically significant. All statistical analyses were performed using SPSS 25 software (IBM, Armonk, NY).

## Results

The clinicopathologic characteristic of all patients are shown in Table 1. Median age was 65 years (range: 51–76 years). Histopathological biopsy was taken before treatment by all patients and reviewed by pathology specialists. Sixteen (52%) patients were diagnosed with squamous cell carcinoma, 9 (29%) patients with adenocarcinoma and 6 (19%) with a non-specified non-small cell lung cancer. Twenty-eight (90.3%) patients had stage III NSCLC according to the 8th UICC TNM Staging System of lung cancer and 3 (9.7%) patients were diagnosed with stage IV NSCLC due to pleural involvement or malignant pleural effusion. All 3 stage IV patients were without sensitizing EGFR or ALK mutations. At diagnosis, 23 (74.2%) patients were heavy smokers (median pack years (PY):40) and 8 (25.8%) patients never smokers.

**Table 1:** patient characteristics

	Number of patients (%)
Age	
≤ 65 years	16 (52)
> 65 years	15 (48)
Gender	
Female	26 (84)
Male	5 (16)
Karnofsky performance status	
> 80%	11 (35)
≤ 80%	20 (65)
UICC stage	
III	28 (90)
IV	3 (10)
T category	
1–2	6 (19)
3–4	25 (81)
N category	
0–1	3 (10)
2–3	28 (90)
Histology	
Squamous cell carcinoma	16 (52)
Non-squamous cell carcinoma	15 (48)
Tobacco consumption (PY)	
0	8 (26)
20–40	8 (26)
> 40	15 (48)
Grading	
Moderately differentiated	2 (6)
Poorly differentiated	27 (87)
anaplastic	2 (6)
TIME	
I	10 (32)
II	5 (16)
III	5 (16)
IV	7 (23)

All patients were treated with definitive concurrent CRT. Twenty-five (81%) patients received platinum-based chemotherapy. A taxane-based combination was applied in 16 (52%) patients. Median biologically equivalent dose (EQD2) to the primary tumor and involved nodes was 65Gy (range: 50-70Gy). Follow-up was conducted as per in-house protocol every 3 months in the first 2 years, every 6 months up to 5 years and afterwards once per year.

The median overall survival in the entire patient collective was 14 months (range: 3–167 months). The 1-year and 2-year OS rates were 67.7 and 19.4%, respectively. The 1 and 2-year actuarial local control rates were 74 and 61%, respectively. Median PFS, 1-year and 2-year PFS were 13 ± 1.4 months, 58 and 19%, respectively.

### Correlations of PD-L1 expression and clinicopathologic characteristics

Correlations of PD-L1 expression and clinicopathologic characteristics are shown in Table 2. PD-L1 inversely correlates with Karnofsky performance status (*p* = 0.023) and positively with CD8+ TIL density (*p* = 0.020).

**Table 2:** Correlations of PD-L1 expression and clinicopathologic characteristics

	Positive, n (%)	Negative, n (%)	*p*-value
Age			
≤ 65 years	8 (50)	8 (50)	
> 65 years	8 (57)	6 (43)	0.834
Gender			
Female	13 (52)	12 (48)	
Male	4 (80)	1 (20)	0.513
Karnofsky performance status			
90–100%	8 (80)	2 (20)	
70–80%	8 (40)	12 (60)	0.023
UICC stage			
III	15 (56)	12 (44)	
IV	1 (33)	2 (67)	0.447
T category			
1–2	4 (67)	2 (33)	
3–4	12 (50)	12 (50)	0.073
N category			
0–1	2 (67)	1 (33)	
2–3	14 (52)	13 (48)	0.402
Histology			
Squamous cell carcinoma	9 (60)	6 (40)	
Non- Squamous cell carcinoma	7 (47)	8 (53)	0.864
Tobacco consumption (PY)			
0	5 (63)	3 (38)	
20–40	3 (38)	5 (63)	
> 40	8 (57)	6 (43)	0.105
Grading			
Moderately differentiated	1 (50)	1 (50)	
Poorly differentiated	14 (54)	12 (46)	
anaplastic	1 (50)	1 (50)	0.223
CD8+ TILs density			
≤ 40%	5 (50)	5 (50)	
> 40%	10 (59)	7 (41)	0.020

### Correlations of CD8+ TIL density and clinicopathologic characteristics

Correlations of CD8+ TIL density and clinicopathologic characteristics are shown in Table 3. CD8+ TIL density inversely correlates with Karnofsky performance status (*p* = 0.038) and positively with PD-L1 expression (*p* = 0.020).

**Table 3:** Correlations of CD8+ TILs density and clinicopathologic characteristics

	high, n (%)	low, n (%)	p-value
Age			
≤ 65 years	12 (80)	3 (20)	
> 65 years	6 (46)	7 (54)	0.403
Gender			
Female	14 (61)	9 (39)	
Male	4 (80)	1 (20)	0.384
Karnofsky performance status			
> 80%	9 (90)	1 (10)	
≤ 80%	9 (50)	9 (50)	0.038
UICC stage			
III	17 (65)	9 (35)	
IV	1 (50)	1 (50)	0.409
T category			
1–2	3 (60)	2 (40)	
3–4	15 (65)	8 (35)	0.751
N category			
0–1	2 (67)	1 (33)	
2–3	16 (64)	9 (36)	0.899
Histology			
Squamous cell carcinoma	8 (62)	5 (39)	
Non- Squamous cell carcinoma	10 (67)	5 (33)	0.681
Tobacco consumption (PY)			
0	6 (75)	2 (25)	
20–40	5 (71)	2 (29)	
> 40	7 (54)	6 (46)	0.11
Grading			
Moderately differentiated	0 (0)	2 (100)	
Poorly differentiated	16 (67)	8 (33)	
anaplastic	2 (100)	0 (0)	0.067
PD-L1 expression			
0%	7 (58)	5 (42)	
≥ 1%	10 (67)	5 (33)	0.02

### Prognostic impact of PD-L1 expression for local control, PFS and OS

Univariate and multivariate analysis for OS, PFS and local control concerning PD-L1 expression are shown in Tables 4, 5 and 6. Univariate analysis for OS showed significance (*p* = 0.048). However, multivariate analysis with cox regression failed (*p* = 0.648). In univariate analysis for PFS and local control, PD-L1 expression was associated with improved PFS (*p* = 0.006) and improved local control rate (*p* = 0.017).

**Table 4:** univariate and multivariate survival analysis

	Survival		*p*-value	
	at 12 months (%)	at 24 months (%)	univariate Analysis	multivariate Analysis
Age				
≤ 65 years	56	19		
> 65 years	80	27	0.676	
Gender				
Female	80	20		
Male	65	23	0.629	
Karnofsky performance status				
> 80%	75	30		
≤ 80%	55	10	0.041	0.077
UICC stage				
III	64	21		
IV	100	33	0.537	
T category				
1–2	67	0		
3–4	68	28	0.395	
N category				
0–1	33	0		
2–3	71	25	0.299	
Histology				
Squamous cell carcinoma	69	25		
Non- Squamous cell carcinoma	67	20	0.935	
Tobacco consumption (PY)				
0	75	25		
20–40	50	12,50		
> 40	73	27	0.758	
Grading				
Moderately differentiated	50	50		
Poorly differentiated	67	19		
anaplastic	100	50	0.758	
PD-L1 expression				
0%	86	29		
≥ 1%	50	19	0.048	0.648
CD8+ TILs density				
≤ 40%	70	40		
> 40%	61	17	0.055	
TIME type				
I	100	60		
II	50	20		
III	71	14		
IV	40	20	0.05	0.048

**Table 5:** univariate and multivariate analysis of local control

	Local control		p-value	
	at 12 months (%)	at 24 months (%)	univariate Analysis	multivariate Analysis
Age				
65 years	58	58		
> 65 years	83	63	0.380	
Gender				
Female	68	57		
Male	80	80	0.941	
Karnofsky performance status				
> 80%	73	67		
≤ 80%	66	33	0.233	
UICC stage				
III	66	62		
IV	100	67	0.862	
T category				
1–2	66	62		
3–4	100	67	0.970	
N category				
0–1	33	33		
2–3	75	64	0.154	
Histology				
Squamous cell carcinoma	70	60		
Non- Squamous cell carcinoma	70	62	0.766	
Tobacco consumption (PY)				
0	83	83		
20–40	51	34		
> 40	72	60	0.417	
Grading				
Moderately differentiated	100	100		
Poorly differentiated	70	59		
anaplastic	50	50	0.487	
PD-L1 expression				
0%	92	79		
≥ 1%	44	44	0.017	0.045
CD8+ TILs density				
≤ 40%	86	75		
> 40%	62	62	0.092	
TIME type				
I	100	80		
II	41	41		
III	83	83		
IV	67	67	0.05	0.694

**Table 6:** univariate and multivariate analysis of progression free survival (PFS)

	PFS		P-value	
	at 12 months (%)	at 24 months (%)	univariate Analysis	multivariate Analysis
Age				
≤ 65 years	50	19		
> 65 years	67	20	0.925	
Gender				
Female	80	20		
Male	54	19	0.868	
Karnofsky performance status				
> 80%	65	25		
≤ 80%	46	9	0.134	
UICC stage				
III	54	18		
IV	100	33	0.458	
T category				
1–2	67	0		
3–4	56	20	0.292	
N category				
0–1	33	0		
2–3	61	20	0.235	
Histology				
Squamous cell carcinoma	56	19		
Non- Squamous cell carcinoma	60	20	0.855	
Tobacco consumption (PY)				
0	63	25		
20–40	38	13		
> 40	67	20	0.633	
Grading				
Moderately differentiated	50	50		
Poorly differentiated	59	15		
anaplastic	50	50	0.831	
PD-L1 expression				
0%	86	29		
≥ 1%	31	13	0.006	0.061
CD8+ TILs density				
≤ 40%	70	30		
> 40%	50	17	0.201	
TIME type				
I	100	60		
II	30	20		
III	71	14		
IV	40	0	0.035	0.144

### Prognostic impact of CD8+ TIL density for local control, PFS and OS

Univariate and multivariate analysis for OS, PFS and local control concerning CD8+ TIL density are shown in Tables 4, 5 and 6. Univariate analysis showed a trend for improved OS and better local control in patients with low CD8+ TIL density (*p* = 0.055; *p* = 0.092).

### Prognostic impact of tumor immunity in the MicroEnvironment (TIME)

According to the Tumor Immunity in the MicroEnvironment (TIME) classification [27, 28], TIME subgroups were evaluated for prognostic outcome for OS, PFS and local control. The longest and shortest OS were achieved in patients with type I (PD-L1^neg^/CD8^low^) and type IV (PD-L1^pos^/CD8^low^) (median OS: 57 ± 37 vs. 10 ± 5 months, *p* = 0.05). In univariate and multivariate analysis for OS, TIME subgroups had significant differences (p = 0.05; *p* = 0.048) as well as in univariate analysis for PFS and local control (p = 0.05; *p* = 0.035).

## Discussion

LA-NSCLC represents a heterogeneous disease which can include large tumor volumes, extensive lymph node involvement and infiltration of the thoracic wall, mediastinum and spine [4–6]. An interdisciplinary strategy is required to define optimal multimodal approaches based on disease stage, patients’ general condition and treatment options according to the latest evidence [29]. The majority of these patients are inoperable due to comorbidities and lymph node involvement. In this situation, multimodal treatment including concurrent application of chemo- and radiotherapy is associated with a moderate toxicity profile and improved patient outcome compared to sequential CRT or radiotherapy alone [7].

Based on the results of the PACIFIC trial, consolidation PD-L1 inhibition with durvalumab is currently considered as standard of care for stage III NSCLC patients without progressive disease following platinum-based concurrent CRT [8, 9]. In stage IV disease, patients with initial TPS ≥ 50% can be offered pembrolizumab monotherapy. Stage IV patient with tumor cell PD-L1 expression < 1% and good PS can receive a combination of platinum-based chemotherapy with PD-1 or PD-L1 inhibition [17–19].

Previous studies suggest that PD-L1 expression can be a potential biomarker for efficacy of NSCLC treatment including surgery, radiotherapy and checkpoint inhibition [19, 21, 30–32]. Retrospective post-hoc analysis of PACIFIC data suggests that outcome of patients appears to depend on initial PD-L1 expression [9, 33].

The principal finding of our study confirms the statement that initial tumor cell PD-L1 expression can be a prognostic factor for inoperable LA-NSCLC treated with concurrent CRT alone. In the study by Vrankar et al., the prognostic relevance of PD-L1 expression was evaluated in 102 patients with stage III NSCLC treated with concurrent chemoradiotherapy [30]. PD-L1 expression ≥5% on tumor cells resulted in significantly unfavorable PFS and OS. However, several limitations of this study need to be taken into account: only a very small patient number (*n* = 7) was considered PD-L1 positive. In addition, negative and unknown states of PD-L1 expression were evaluated together. In our study, 52% of all patients were considered PD-L1 positive according to the cut-off value in the PACIFIC trial.

Data of the predictive value of PD-L1 expression on tumor cells in combination with CD8+ tumor-infiltrating lymphocyte (TIL) density in patients with locally advanced NSCLC is limited [34, 35]. Tokito et al. found CD8+ TIL density is an independent prognostic factor for OS [34]. Interestingly, PD-L1 expression (≥5%) on tumor cells has shown no prognostic role in this study in contrast to previous reports [19, 32, 36]. Indeed, patients with low or no PD-L1 expression on tumor cells could respond to PD-1/PD-L1 inhibition as well and show a durable response [22, 37]. In addition, PD-L1 expression can vary between tumor cells, surrounding non-malignant tissue and peripheral immune cells [38–40]. Treatment modality appears to have an impact on PD-L1 expression [41, 42]. Fujimoto et al. evaluated PD-L1 expression on tumor cells before and after CRT and found that alteration of PD-L1 expression was associated with survival in patients with LA-NSCLC [42].

As a result, the interaction of tumor and immune cells in the treatment and immune response is still poorly understood. Based on preclinical and clinical data, the involvement of CD8+ TILs plays a crucial role in tumor-associated immune response [43]. The CD8+ TIL density in the tumor microenvironment has been suggested to predict the oncologic outcome in different cancer types such as colorectal cancer, malignant melanoma and anal cancer [28, 44, 45]. Based on previous studies, four different types of tumor immune microenvironment have been identified according to PD-L1 expression of tumor cells and presence or absence of TILs in the tumor microenvironment. These included type I (PD-L1^neg^ with no TILs indicating immune ignorance), type II (PD-L1^pos^ with TILs implying adaptive immune resistance, type III (PD-L1^neg^ with TILs suggesting a role of other suppressor(s) in promoting immune tolerance) and type IV (PD-L1^pos^ with no TILs indicating intrinsic induction). In our study, the longest OS was achieved in patients with type I (PD-L1^neg^/CD8^low^) in contrast to previous studies investigating the prognostic value of PD-L1 expression combined with CD8+ TIL density. In the studies of Tokito et al. and El-Guindy et al., patients with PD-L1^neg^/CD8^high^ had the longest OS and according to Yang et al. the patient subgroup with PD-L1^pos^/CD8^high^ showed the longest OS [34, 35, 46]].

The shortest OS in our study was seen in patients with type IV (PD-L1pos/CD8low) and well in accordance with the published literature [33, 34, 45]. This finding could be explained by the lack of immune-mediated tumor response. Tumor cells can decrease their immunogenicity through interaction of PD-L1 with PD-1 on T cells. As a result, the tumor can evade the immune surveillance. In addition, a lack of CD8+ TILs can account for most non-responders to PD-1/PD-L1 inhibition [28].

Several limitations of this study need to be considered when interpreting the results. Firstly, the retrospective nature of this study and the possibility of unknown biases. Secondly, the relatively small number of patients included in the analysis and lastly, all patients were treated at a single center. However, we are convinced that our findings supporting the assessment of CD8+ TIL density combined with PD-L1 expression, instead of PD-L1 expression alone is of important clinical relevance and requires special consideration in future trials.

## Conclusion

Initial PD-L1 expression on tumor cells can be a prognostic factor for local control, PFS and OS and **correlates** with CD8+ TILs density in inoperable LA-NSCLC. Assessment of PD-L1 expression in combination with CD8+ TILs density, instead of PD-L1 expression alone, appears to be of strong prognostic relevance in patients treated with concurrent CRT. Future prospective studies are warranted to verify our findings.

## Data Availability

The datasets used and analysed during the current study are available from the corresponding author on reasonable request.
